# Supplementary material for: Discrete energy levels of Caroli-de Gennes-Matricon states in quantum limit in FeTe0.55Se0.45
Source: Nat Commun. 2018 Mar 6;9:970. doi: 10.1038/s41467-018-03404-8 (PMC5840178; doi:10.1038/s41467-018-03404-8)
Supplement: Supplementary file 1 — Supplementary Information [file 41467_2018_3404_MOESM1_ESM.pdf]

Discrete energy levels of Caroli-de Gennes-Matricon states in quantum limit in $FeTe_{0.55}Se_{0.45}$

Chen *et al.*

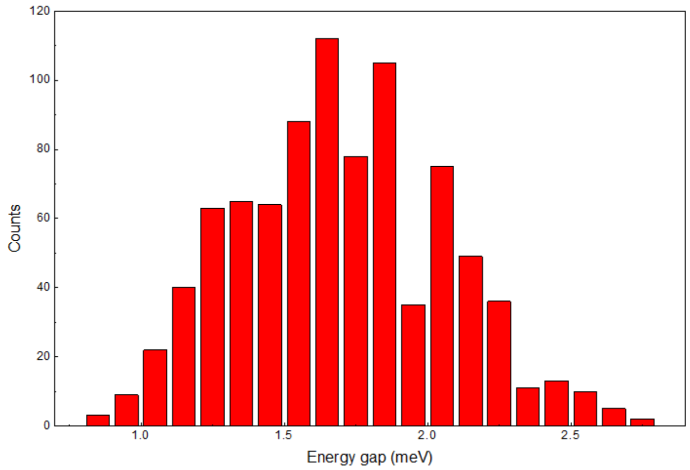

Supplementary Figure 1 | Histogram of the superconducting gap *Δ* at 0T. The absolute values of superconducting gaps are determined by the coherence-peak positions for all the 336 spectra measured on one sample. The typical spectra are shown in Fig. 1c. There are usually one or two pairs of coherence peaks with peak energies ranging from about ±1.1 mV to ±2.1 mV.

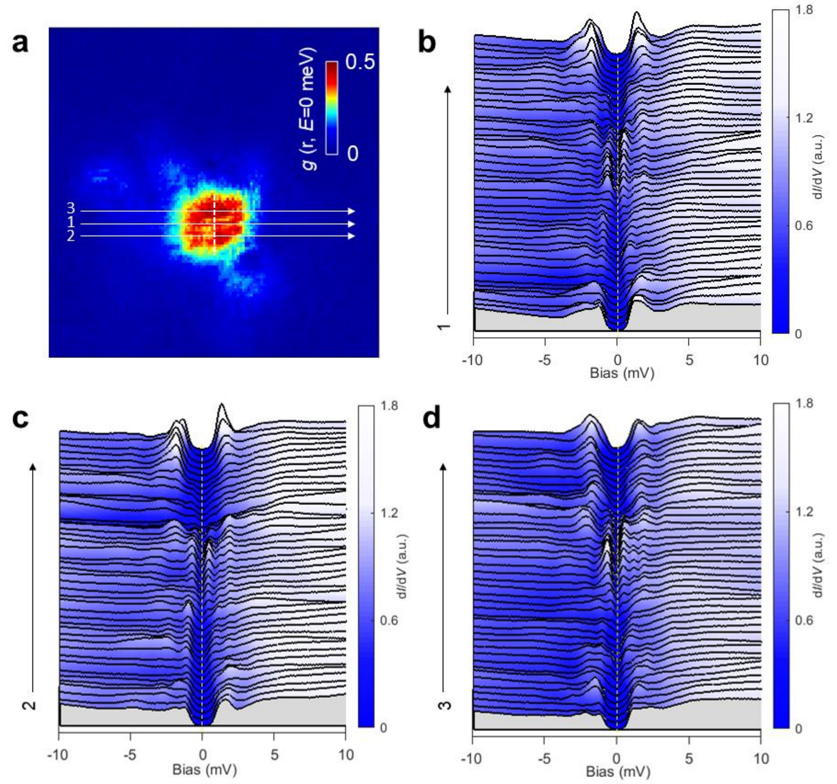

Supplementary Figure 2 | Vortex image and CdGM states along three horizontal lines. **a**, Image of the same vortex as in Fig. 2. **b**-**d**, Tunnelling spectra measured along the white lines marked from 1 to 3 in **a**. The density of the measuring points for each curve is twice than that in Fig. 2, i.e., the increment step is 3.8 Å between two neighboring points. The dashed lines in **b**-**d** show the positions of zero bias for each figure.

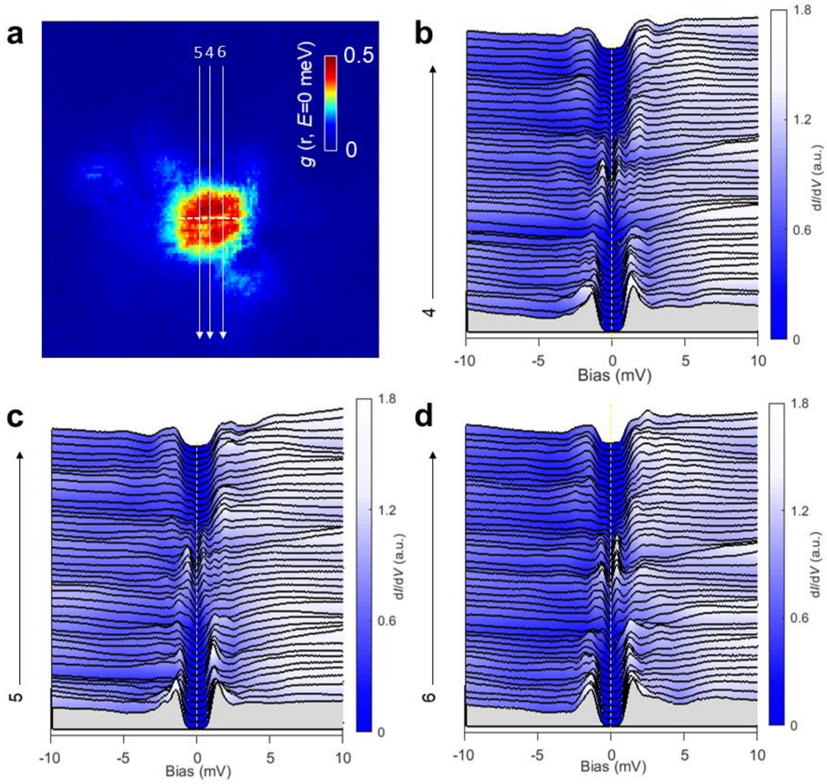

Supplementary Figure 3 | Vortex image and CdGM states along three vertical lines. **a**, Image of the same vortex as in Fig. 2 in the main text. **b**-**d**, Tunnelling spectra measured along vertical white lines marked from 4 to 6 in **a** with increment steps of 3.8 Å. The dashed lines in **b**-**d** show the positions of zero bias voltage for each figure.

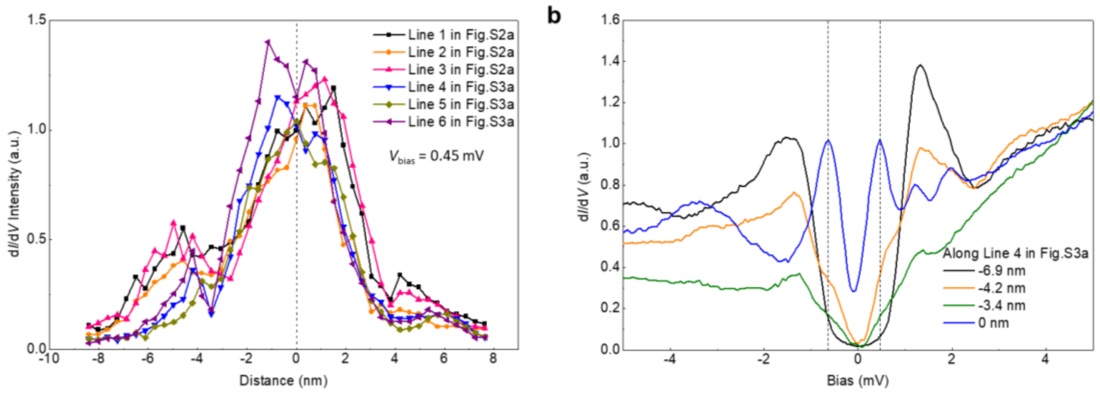

Supplementary Figure 4 | Possible second-order vortex bound state peak of $E_{1/2}$. a, Spatial evolution of the d*I*/d*V* intensity taken at a fixed bias voltage of $E_{1/2}$ = 0.45 mV. One can find possible second-order peaks locating at about ±4.5 nm. **b**, Tunnelling spectra measured at some typical positions. The blue curve shows the spectrum at the vortex core center, which exhibits the clear $E_{\pm 1/2}$ peaks. The vertical dashed lines show the $E_{\pm 1/2}$ peak positions. The yellow curve shows two kinks at similar energies on the spectrum measured at -4.2 nm away from the vortex center.

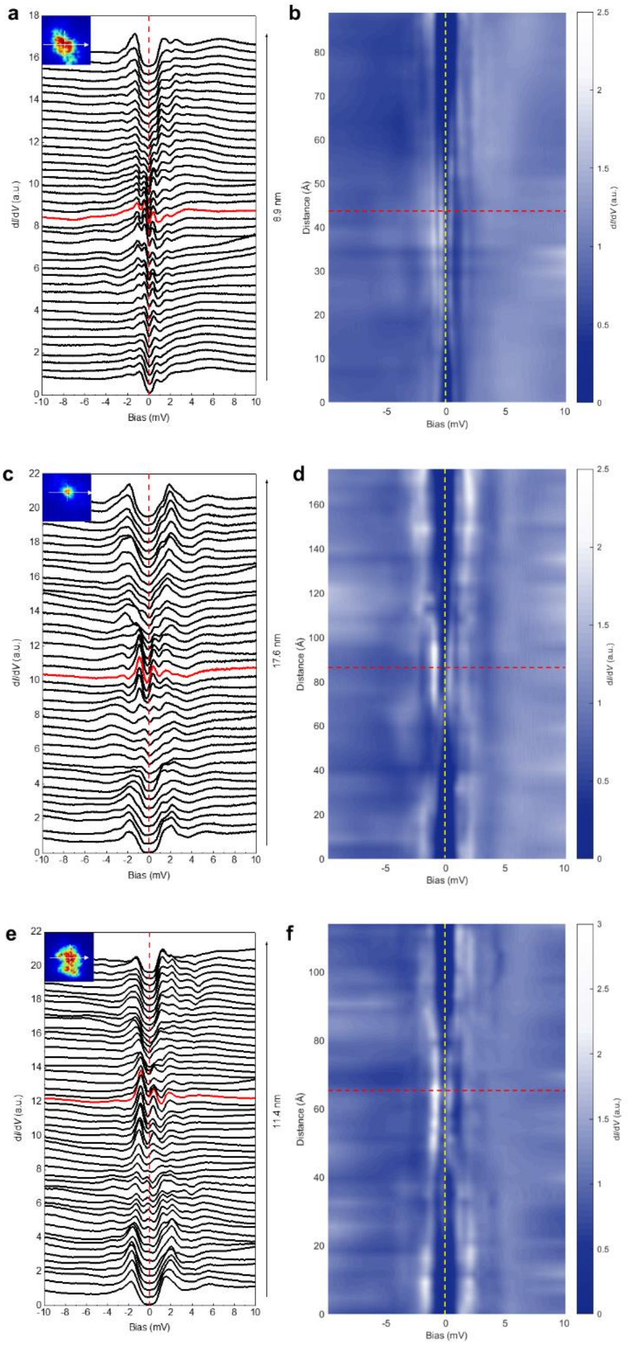

Supplementary Figure 5 | Other types of vortex core states in different vortices. a,**c**,**e**, Series of spatially resolved tunnelling spectra measured at 0.48 K and 4 T along the traces marked by the white lines drawn in the insets. The red lines in **a**, **c**, and **e** represent the spectra measured at vortex core center. **b**,**d**,**f** Colour plots of spatial profile of the spectra shown in **a**, **c**, and **e**, respectively. The vortex bound states in **a**-**d** exhibit as two asymmetric peaks to zero-bias, while the bound state in **e** and **f** shows as a single peak with peak position at some positive energy.

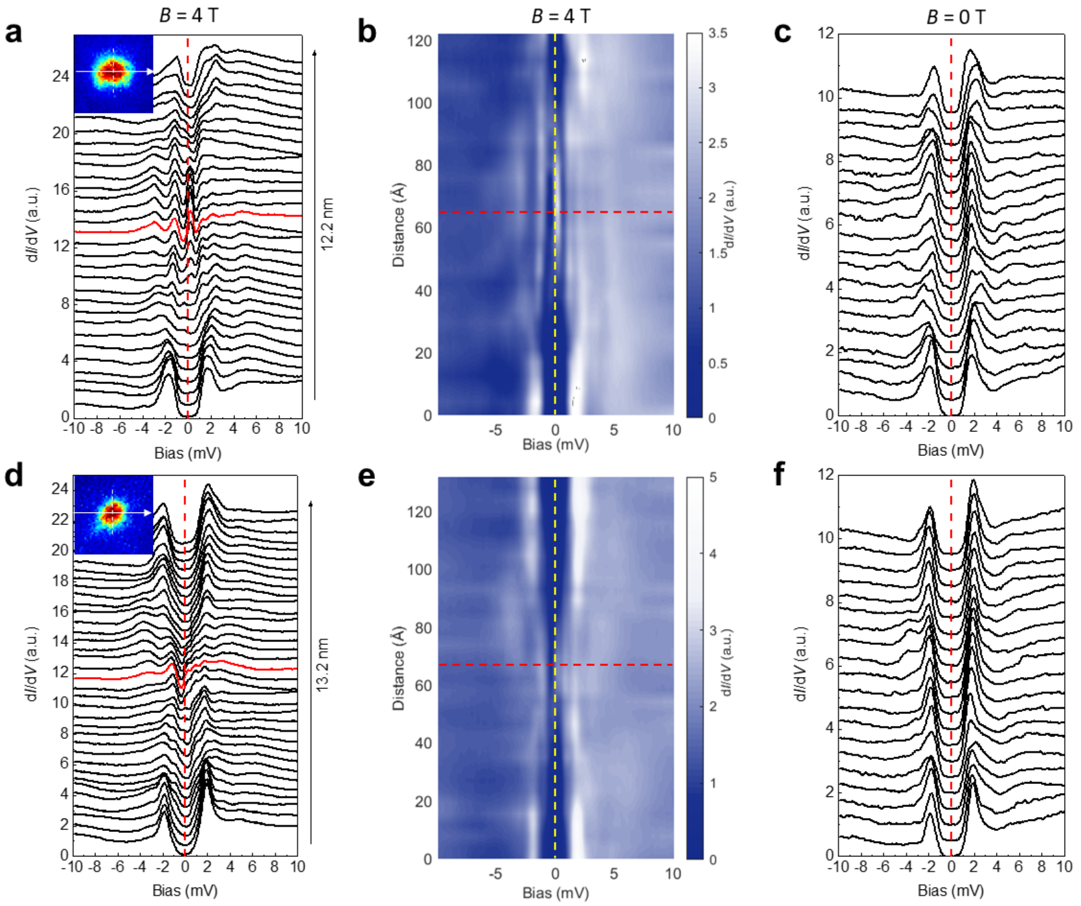

Supplementary Figure 6 | Other vortices with three bound state peaks on the positive energy side. a,**b**, Series of spatially resolved tunnelling spectra and the corresponding 2D colour plot of the spectra measured across a vortex with three bound state peaks at 0.48 K and 4 T along the traces marked by the white arrowed lines drawn in the inset of **a**. **c**, Spatial evolution of tunneling spectra at 0.4 K without magnetic field in the same region where the vortex would appear under a magnetic field. **d**-**f**, Data taken on another vortex, with the same descriptions as **a-c**, respectively.

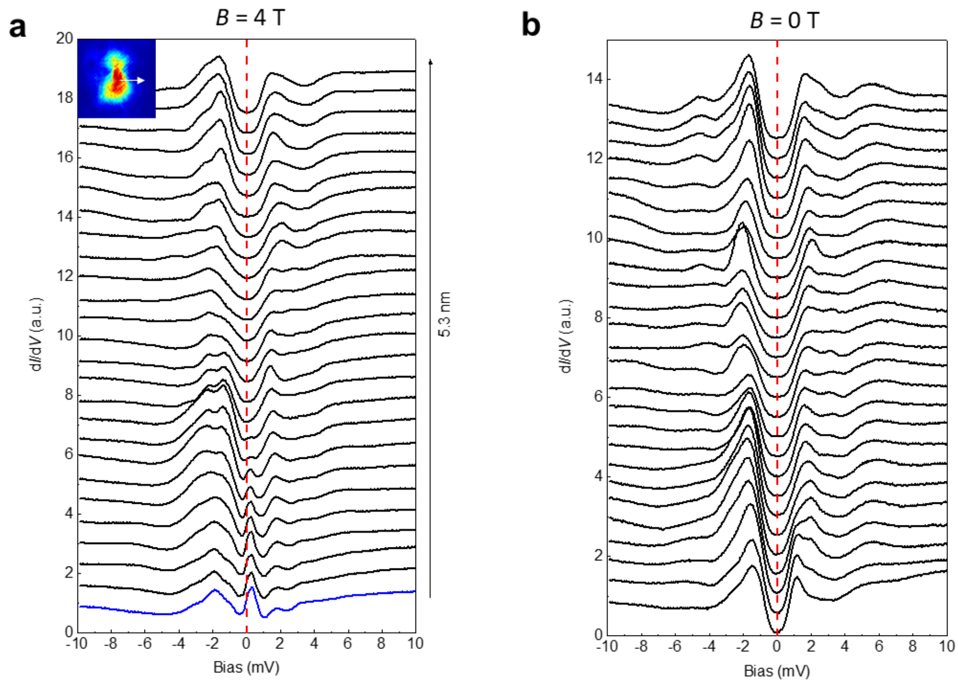

Supplementary Figure 7 | Spatial evolution of tunneling spectra measured at 4 T and 0 T for the vortex with a dominant peak at positive bias. a, A set of spatially resolved tunnelling spectra which are the same as those shown in Fig. 4c. **b**, Spatial evolution of tunnelling spectra at 0.4 K without magnetic field along the same arrowed line where the vortex would appear under a magnetic field.

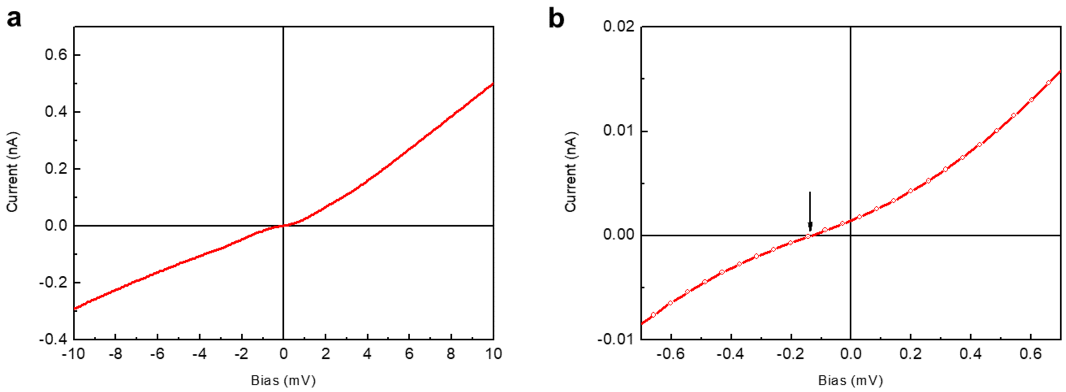

Supplementary Figure 8 | Bias-offset calibration from averaged *I-V* curve. a, Average *I-V* curves for all the spectra measured along line 3 in Supplementary Fig. 2. **b**, Enlarged view of the same curve near zero-bias.

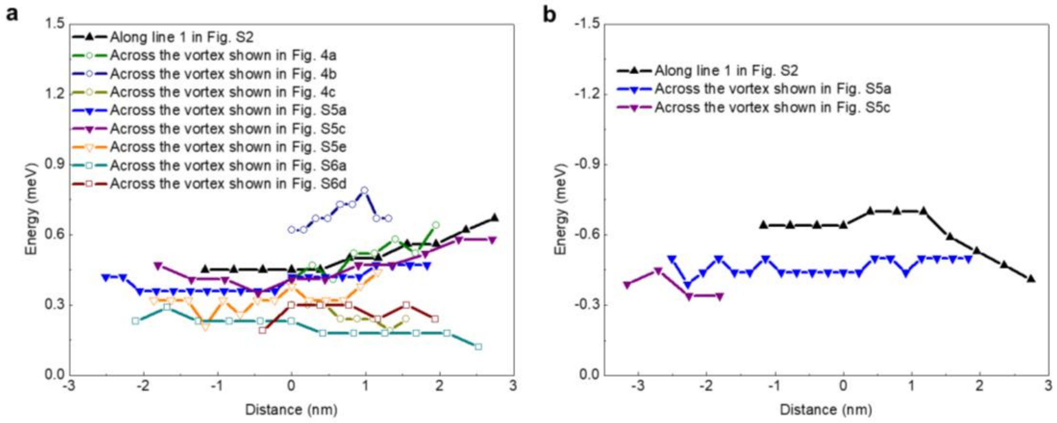

Supplementary Figure 9 | Distribution of the lowest bound state energies for different vortices. The data represent the spatial evolution of the lowest bound state energies for positive bias (possible $E_{+1/2}$ energy, shown in **a**) and negative bias (possible $E_{-1/2}$ energy, shown in **b**). The open symbols represent the peak positions for the single asymmetric bound state peaks locating at some positive energies, while the solid symbols show the data for the CdGM states existing on both positive and negative bias voltages. The absolute values of the peak positions are usually larger for $E_{-1/2}$ peaks than $E_{+1/2}$ peaks.

Supplementary Note 1 | Further information of CdGM states in the vortex shown in Fig. 2

Supplementary Fig. 4a shows the spatial evolution of the differential conductance amplitude measured at a fixed energy of $E_{1/2}$ = 0.45 mV. One can find that a pair of small second peaks appears at about ± 4.5 nm. Supplementary Fig. 4b shows some spectra measured at some typical positions. One can find clear kinks near 0.45 mV on the spectrum measured at -4.2 nm away from the vortex core center. One possible reason for these small dI/dV peaks come from the amplitude oscillations as a function of distance away from the vortex center. Theoretically, the peak amplitude of DOS for one selected bound state will show spatial oscillation in quantum limit[1,2]. The space period of the amplitude oscillation is usually related to $1/k_F$. In conventional superconductors, the Fermi energy E_F or the Fermi vector k_F is much larger than Δ or $1/\xi_0$. Hence, the second-order bound state peak of $E_{1/2}$ exists at somewhere $r_{2nd} < \xi_0$, e.g., $r_{2nd} \approx 0.5\xi_0 = 4/k_F$ for a material with $k_F\xi_0 = 8$ from the theoretical calculation[1]. However, k_F is very small in $FeTe_{0.55}Se_{0.45}$, i.e., $k_F\xi_0 \approx 1.7$ to 3. In this case, it is not strange that the second order of $E_{1/2}$ bound state peak exists at the place $r_{2nd} > \xi_0 \approx 25$ Å. We argue that the small dI/dV peaks locating at about ± 4.5 nm is from the amplitude oscillation of CdGM state in quantum limit, which is another proof for this state.

Supplementary Note 2 | Offset-bias calibration in tunnlling spectra measurements

The offset of the tunnelling current is about several pico-amperes, and it is calibrated in the tip-withdraw status. In such condition, the distance between the tip and sample is about 100 nm, and the tunnelling current in this situation should be zero approximately. Following this method, the offset of tunneling current was calibrated before the measurements. Then the bias offset in our experiment is determined from the averaged I-V curves, i.e., we use the voltage value for zero tunnelling current as

the offset voltage, which means that the current should be zero when the effective bias-voltage is zero. One example is shown in Supplementary Fig. 8, and the offset determined from the enlarged view of the averaged *I-V* curves is about -0.13 mV. The bias-offset values for zero-tunnelling-currents are -0.15, -0.13, and -0.10 meV for the spectra shown in Fig. 4a, b, and c respectively.

Supplementary References

1. Hayashi, N., Isoshima, T., Ichioka, M. & Machida, K. Low-lying quasiparticle excitations around a vortex core in quantum limit. *Phys. Rev. Lett.* **80**, 2921-2924 (1998).

2. Kaneko, S. *et al.* Quantum limiting behaviors of a vortex core in an anisotropic gap superconductor YNi_2B_2C. *J. Phys. Soc. Jpn.* **81**, 063701 (2012).
